# Modeling of the Progressive Degradation of the Nigrostriatal Dopaminergic System in Mice to Study the Mechanisms of Neurodegeneration and Neuroplasticity in Parkinson’s Disease

**DOI:** 10.3390/ijms24010683

**Published:** 2022-12-30

**Authors:** Anna Kolacheva, Alyona Bannikova, Ekaterina Pavlova, Vsevolod Bogdanov, Michael Ugrumov

**Affiliations:** Koltzov Institute of Developmental Biology of the Russian Academy of Sciences, 26 Vavilova Street, 119334 Moscow, Russia

**Keywords:** Parkinson’s disease, models of Parkinson’s disease, neurodegeneration, neuroplasticity, dopamine, dopaminergic neurons, nigrostriatal system, striatum, substantia nigra, 1-methyl-4-phenyl-1,2,3,6-tetrahydropyridine, mice

## Abstract

The fight against neurodegenerative diseases, including Parkinson’s disease (PD), is among the global challenges of the 21st century. The low efficiency of therapy is due to the late diagnosis and treatment of PD, which take place when there is already significant degradation of the nigrostriatal dopaminergic system, a key link in the regulation of motor function. We have developed a subchronic mouse model of PD by repeatedly administering 1–methyl–4–phenyl–1,2,3,6–tetrahydropyridine (MPTP) at gradually increasing doses with a 24 h interval between injections, a period comparable to the time of MPTP metabolism and elimination from the body. This model reproduces the main hallmarks of PD: progressive degeneration of dopaminergic neurons; the appearance of motor disorders with a 70–80% decrease in the level of dopamine in the striatum; an increase in dopamine turnover in the striatum to compensate for dopamine deficiency. When comparing the degradation of the nigrostriatal dopaminergic system and motor disorders in mice in the acute and subchronic models of PD, it has turned out that the resistance of dopaminergic neurons to MPTP increases with its repeated administration. Our subchronic model of PD opens up broad prospects for studying the molecular mechanisms of PD pathogenesis and developing technologies for early diagnosis and preventive treatment.

## 1. Introduction

One of the global challenges of the 21st century is the fight against socially significant incurable neurodegenerative diseases, primarily Alzheimer’s disease and Parkinson’s disease (PD). The low effectiveness of the treatment of these diseases is due to the fact that they are diagnosed by the appearance of characteristic clinical symptoms and begin to be treated only many years (up to 30 years) after the onset of the disease [1,2]. A key link in the pathogenesis of PD, which is the subject of this study, is the death of dopaminergic (DAergic) neurons in the nigrostriatal system of the brain responsible for the regulation of motor function [3]. By the time the first motor symptoms appear in patients, 50–60% of DAergic neurons localized in the substantia nigra (SN) of the brain die, and the level of dopamine (DA) in the striatum, the site of projection of the DAergic axons, decreases by 70–80% [2,4,5,6,7,8]. It is noteworthy that it is in the striatum that DA, as a neurotransmitter, plays a key role in the regulation of motor function [9,10,11].

Based on the above data and ideas, the current strategy for combating PD aims to establish an early diagnosis long before the onset of motor symptoms, at the so-called preclinical stage, as well as to develop preventive neuroprotective treatment [12]. Neuroprotective treatment should focus, on the one hand, on preventing or at least slowing down the death of DAergic neurons, and, on the other hand, on activating compensatory processes that prevent motor disorders at the preclinical stage. Such treatment will significantly prolong the preclinical stage, and, consequently, the period of normal social and physical activity of patients. In accordance with the paradigm of translational medicine, the development of such technologies should be based on fundamental knowledge about the molecular mechanisms of PD pathogenesis at various stages of development [2].

Given that the opportunities for obtaining biological samples from patients diagnosed with PD at the so-called clinical stage are extremely limited, and that this is impossible at the preclinical stage, the vast majority of studies of the molecular mechanisms of PD pathogenesis are carried out on experimental models. This means that success in developing new technologies for diagnosing and treating PD largely depends on how and with what accuracy the pathogenesis of this disease will be reproduced in experimental models. This mostly explains the large number of publications devoted to the development of PD models: acute, subchronic, and chronic [13,14,15,16,17,18,19,20,21,22,23,24,25,26,27,28,29,30,31,32,33,34,35,36,37]. The number of these publications continues to grow, as no single model is able to reproduce any pathology fully, including PD. The aim of this study has been to develop in mice a subchronic model of the progressive development of PD using 1-methyl-4-phenyl-1,2,3,6-tetrahydropyridine (MPTP). This model should reproduce PD pathogenesis from the onset of the preclinical stage to the clinical stage. The transition from the first of these stages to the second should occur with a threshold decrease in the level of DA in the striatum by 70–80%, which should be accompanied by the appearance of motor disorders. In the future, this model can be used to study the molecular mechanisms of the pathogenesis of PD and to develop early diagnosis and preventive treatment of this severe disease.

## 2. Results

### 2.1. The Motor Behavior of Mice before and after the Administration of 1-Methyl-4-phenyl-1,2,3,6-tetrahydropyridine

**Figure 1:**
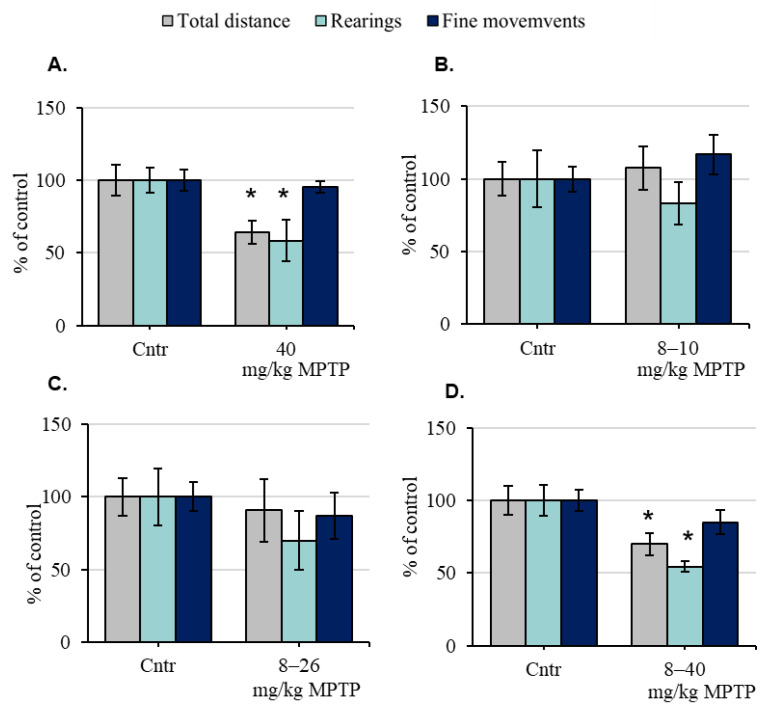
The motor behavior of mice in the open field test, assessed by the total distance, number of rearings, and number of fine movements in % of the control after 24 h: after a single injection of 1-methyl-4-phenyl-1,2,3,6-tetrahydropyridine (MPTP) at a dose of 40 mg/kg (**A**), after successive MPTP injections at doses of 8 and 10 mg/kg (**B**), after successive MPTP injections at doses of 8, 10, 12, 16, 20, and 26 mg/kg (**C**), and after successive MPTP injections at doses of 8, 10, 12, 16, 20, 26, and 40 mg/kg (**D**) (“n” per group = 10–12). * *p* < 0.05, significant differences compared with the control, taken as 100% (the Student’s *t*-test). Data are presented as mean ± SEM. Cntr—the control group received 0.9% NaCl.

Twenty-four hours after a single administration of MPTP at a dose of 40 mg/kg, the total distance and number of rearings in mice were reduced by 36% and 42%, respectively, compared with the control, taken as 100% (Figure 1A). The number of fine movements did not change compared with the control.

Twenty-four hours after two MPTP injections at doses of 8 and 10 mg/kg and after six MPTP injections at doses from 8 to 26 mg/kg, the motor behavior parameters of mice did not change compared with the control (Figure 1B,C). Twenty-four hours after seven MPTP injections at increasing doses from 8 mg/kg to 40 mg/kg, the total distance and number of rearings in mice were reduced by 30% and 46%, respectively. The number of fine movements was maintained at the control level (Figure 1D).

### 2.2. The Concentration of Dopamine and Its Metabolites in the Striatum of Mice after the Administration of 1-Methyl-4-phenyl-1,2,3,6-tetrahydropyridine

**Figure 2:**
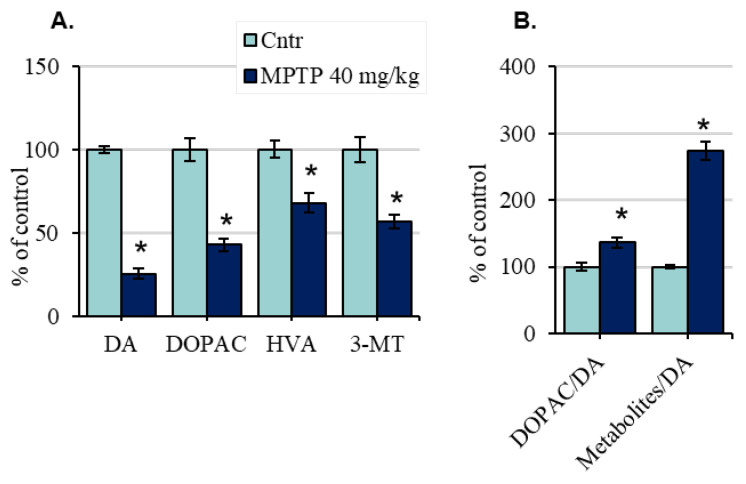
Dopamine (DA), 3,4-dihydroxyphenylacetic acid (DOPAC), homovanillic acid (HVA), and 3-methoxytyramine (3-MT) concentrations (**A**) and dopamine turnover: DOPAC/DA or Metabolites/DA((DOPAC + HVA + 3-MT)/DA) (**B**), presented as % of the control, in the striatum of mice 24 h after a single injection of 1-methyl-4-phenyl-1,2,3,6-tetrahydropyridine (MPTP) at a dose of 40 mg/kg) (“n” per group = 8). * *p* < 0.05, significant differences compared with the control, taken as 100% (the Student’s *t*-test). Data are presented as mean ± SEM. Cntr—the control group received 0.9% NaCl.

In mice of the 1st group, 24 h after a single injection of MPTP at a dose of 40 mg/kg, the concentration of DA decreased by 74% compared with the control (Figure 2A, Table S1). The concentrations of 3,4-dihydroxyphenylacetic acid (DOPAC), homovanillic acid (HVA), and 3-methoxytyramine (3-MT) were reduced by 57%, 32%, and 43% compared to the control, respectively (Table S1). DA turnover, as a DOPAC/DA ratio, increased by 37%, and the (DOPAC + HVA + 3-MT)/DA ratio increased by 174% (Figure 2B).

**Figure 3:**
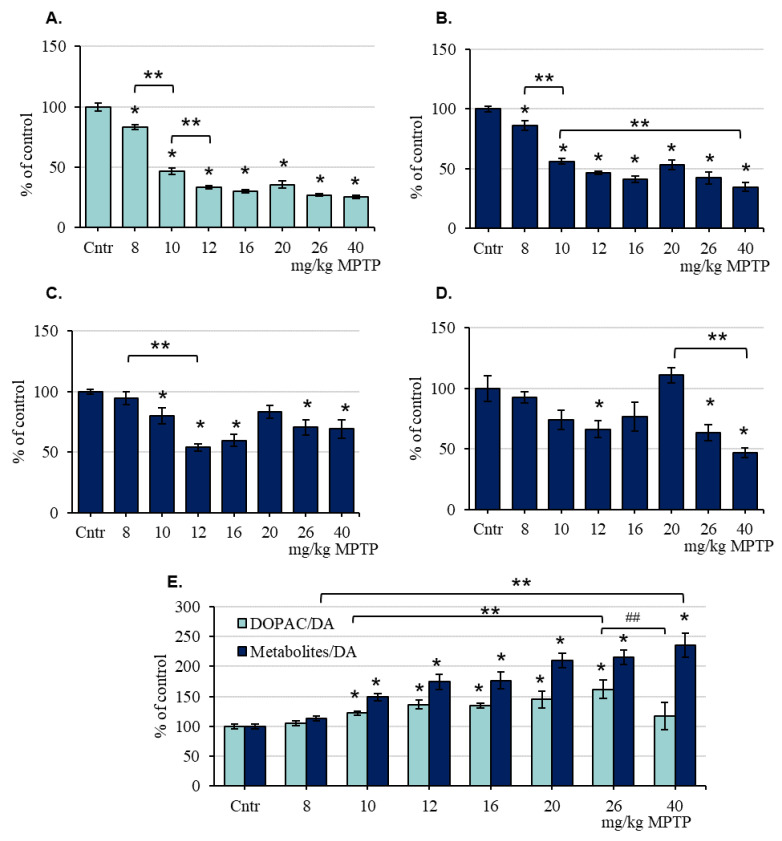
The concentrations of dopamine (DA) (**A**), 3,4-dihydroxyphenylacetic acid (DOPAC) (**B**), homovanillic acid (HVA) (**C**), and 3-methoxytyramine (3-MT) (**D**), as well as dopamine turnover: DOPAC/DA or Metabolites/DA ((DOPAC + HVA + 3-MT)/DA) (**E**), presented as % of the control, in the striatum of mice 24 h after each of the successive injections of 1-methyl-4-phenyl-1,2,3,6-tetrahydropyridine (MPTP) at increasing doses from 8 to 40 mg/kg (“n” per group = 6–8). * *p* < 0.05, significant differences compared with the control, taken as 100% (the Student’s *t*-test). ** *p* < 0.05 significant differences and tendency to change (## *p* < 0.1) between selected parameters (one-way ANOVA). Data are presented as mean ± SEM. Cntr—the control group received 0.9% NaCl.

In mice of the 2nd group (increasing MPTP doses from 8 to 40 mg/kg), the DA concentration in the striatum of the control mice for each subsequent MPTP injection was the same: on average, 105.41 ± 1.69 pmol/mg (Table S1). After the 1st MPTP injection (8 mg/kg), the DA concentration decreased by 17%; after the 2nd injection (10 mg/kg), by 53.1%; and after the 3rd injection (12 mg/kg), by 66.6%. Subsequent MPTP injections at doses of 16, 20, 26, and 40 mg/kg did not cause any statistically significant changes in DA concentration compared with its level after the 3rd MPTP injection (Figure 3A, Table S1).

In mice of the 2nd group, the concentrations of DOPAC, HVA, and 3-MT in the striatum of the control mice for each subsequent MPTP injection were the same: on average, 6.33 ± 0.4 pmol/mg, 12.0 ± 0.6 pmol/mg, and 2.0 ± 0.2 pmol/mg, respectively (Table S1). After the 1st MPTP injection (8 mg/kg), the concentration of DOPAC in the striatum decreased by 14%; after the 2nd injection (10 mg/kg), by 44%; and after the 7th, by 66% (Figure 3B, Table S1). The concentration of HVA in the striatum of mice did not change after the 1st MPTP injection (8 mg/kg); after the 2nd injection (10 mg/kg), it decreased by 20% compared with the control; after the 3rd injection (12 mg/kg), it decreased by 46%; after the 4th injection (16 mg/kg), it remained at the same level; after the 5th injection, it increased to the control level; after the 6th injection, it decreased by 29% and remained at this level after the 7th injection (Figure 3C, Table S1). The concentration of 3-MT in the striatum of mice for the first time decreased by 34% compared with the control after the 3rd MPTP injection (12 mg/kg). After the 4th (16 mg/kg) and 5th (20 mg/kg) MPTP injections, the 3-MT concentration increased to the control level. After the 6th and 7th MPTP injections, the concentration of 3-MT in the striatum decreased by 36% and 53%, respectively, compared with the control (Figure 3D, Table S1).

After determining the concentrations of DA and its metabolites in mice of the 2nd group, DA turnover was calculated using the two previously mentioned methods: in the first case, as the ratio of the DOPAC concentration to the DA concentration, and in the second case, as the ratio of the sum of the concentrations of all metabolites (DOPAC, HVA, and 3-MT) to the DA concentration (Figure 3E). Using both calculations, DA turnover in the control and after the first MPTP injection (8 mg/kg) was the same. After the second MPTP injection (10 mg/kg), according to both calculations, DA turnover increased relative to the control and did not change until the 6th MPTP injection (26 mg/kg). After the last, 7th MPTP injection (40 mg/kg), DA turnover calculated with respect to all metabolites did not change compared with that after the 6th injection, but DA turnover calculated as the ratio of the DOPAC concentration to the DA concentration returned to the control level (Figure 3E). The difference in the ratio of DOPAC/DA after the 6th and 7th injections of MPTP tended to change (*p* = 0.0833 by one-way ANOVA).

### 2.3. The Content of Dopamine and Its Metabolites in the Substantia Nigra of Mice after the Administration of 1-Methyl-4-phenyl-1,2,3,6-tetrahydropyridine

**Figure 4:**
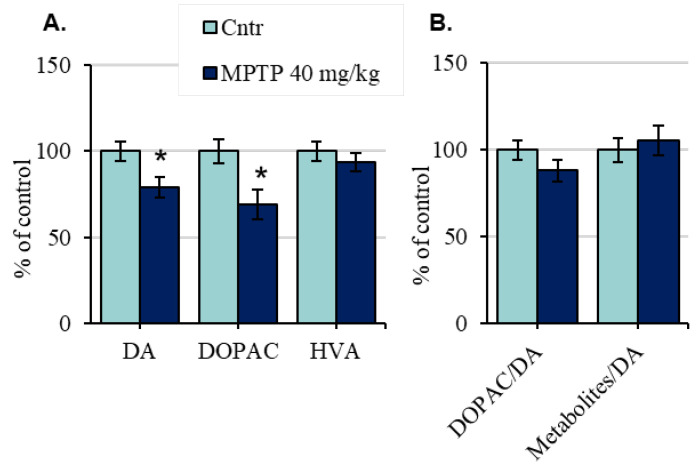
The content of dopamine (DA), 3,4–dihydroxyphenylacetic acid (DOPAC), and homovanillic acid (HVA) (**A**), as well as DA turnover, calculated as DOPAC/DA or Metabolites/DA ((DOPAC + HVA)/DA) (**B**), presented as % of the control, in the substantia nigra of mice 24 h after a single injection of 1-methyl-4-phenyl-1,2,3,6-tetrahydropyridine (MPTP) at a dose of 40 mg/kg. * *p* < 0.05, significant differences compared with the control, taken as 100% (the Student’s *t*-test). n = 8. Data are presented as mean ± SEM. Cntr—the control group received 0.9% NaCl.

In mice of the 1st group, the content of DA in the SN 24 h after a single MPTP injection at a dose of 40 mg/kg decreased by 21% compared with the control (Figure 4A, Table S2). The content of DOPAC was reduced by 31%, and the content of HVA did not change (Figure 4A, Table S2). DA turnover calculated relative to DOPAC or to all DA metabolites (DOPAC + HVA) similarly did not change (Figure 4B).

**Figure 5:**
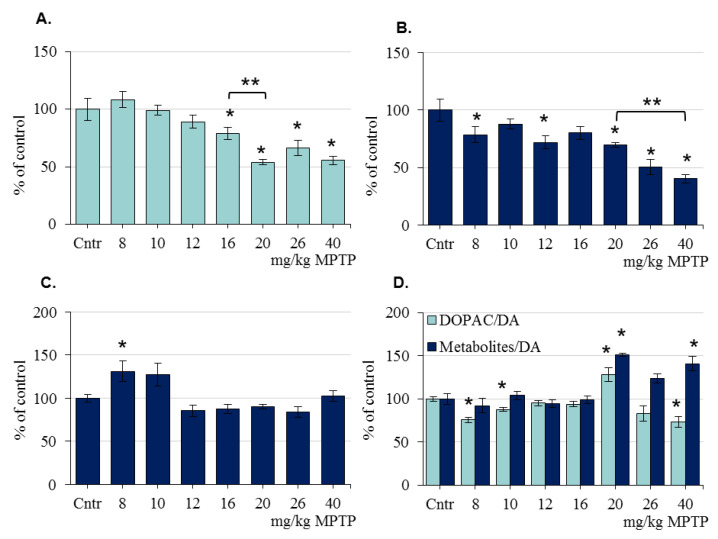
The content of dopamine (**A**), 3,4-dihydroxyphenylacetic acid (DOPAC) (**B**), and homovanillic acid (HVA) (**C**), as well as dopamine turnover, calculated as DOPAC/DA or Metabolites/DA ((DOPAC + HVA)/DA) (**D**), presented as % of the control, in the substantia nigra of mice 24 h after each of the successive injections of 1-methyl-4-phenyl-1,2,3,6-tetrahydropyridine (MPTP) at increasing doses from 8 to 40 mg/kg (“n” per group = 6–8). * *p* < 0.05, significant differences compared with the control, taken as 100% (the Student’s *t*-test). ** *p* < 0.05, significant differences between the selected parameters (one-way ANOVA). Data are presented as mean ± SEM. Cntr—the control group received 0.9% NaCl.

In mice of the 2nd group, the DA content in the SN remained at the control level after the first three MPTP injections: at doses of 8, 10, and 12 mg/kg, respectively (Figure 5A, Table S2). After the 4th administration of MPTP (16 mg/kg), the content of DA decreased by 21% compared with the control, and after the 5th administration of MPTP (20 mg/kg), it declined by 46%. Subsequent MPTP injections did not result in changes in DA content compared with the level reached after administering MPTP at a dose of 20 mg/kg (Figure 5A).

The DOPAC content in the SN was reduced by 21–30% compared with the control 24 h after MPTP was injected at doses of 8, 12, 20, and 26 mg/kg, (Figure 5B, Table S2). Only after the 7th MPTP injection (40 mg/kg) did the DOPAC content decrease even more: by 60% (Figure 5B). The content of HVA in the SN increased by 31% after the 1st MPTP injection (8 mg/kg), while all subsequent doses did not change the content of HVA compared with the control, up to the 7th MPTP injection at a dose of 40 mg/kg (Figure 5C, Table S2).

DA turnover in the SN, calculated as the DOPAC/DA ratio, was reduced compared with the controls after the 1st (8 mg/kg) and 2nd (10 mg/kg) MPTP injections, by 24% and 12%, respectively (Figure 5D). Subsequent MPTP injections up to a dose of 16 mg/kg did not lead to a change in this indicator. After administering MPTP at a dose of 20 mg/kg, this indicator increased; after injecting MPTP at a dose of 26 mg/kg, the DOPAC/DA ratio returned to the control level; and after the 7th MPTP injection (40 mg/kg), DA turnover was reduced by 26% in relation to the control (Figure 5D). DA turnover, calculated as the ratio of the sum of the content of DOPAC and HVA to the content of DA, did not change compared with the control after the administration of increasing MPTP doses up to 16 mg/kg inclusively, while after 20 mg/kg, a 50% increase in this indicator was shown, and after injecting MPTP at a dose of 26 mg/kg, this indicator returned to the control level (*p* = 0.06). After the 7th MPTP injection (40 mg/kg), DA turnover, calculated as the ratio of the sum of the DOPAC and HVA content to the DA content, increased by 41% relative to the control (Figure 5D).

### 2.4. The Content of Tyrosine Hydroxylase (TH)-Immunopositive Neurons in the Substantia Nigra of Mice after the Administration of 1-Methyl-4-phenyl-1,2,3,6-tetrahydropyridine

**Figure 6:**
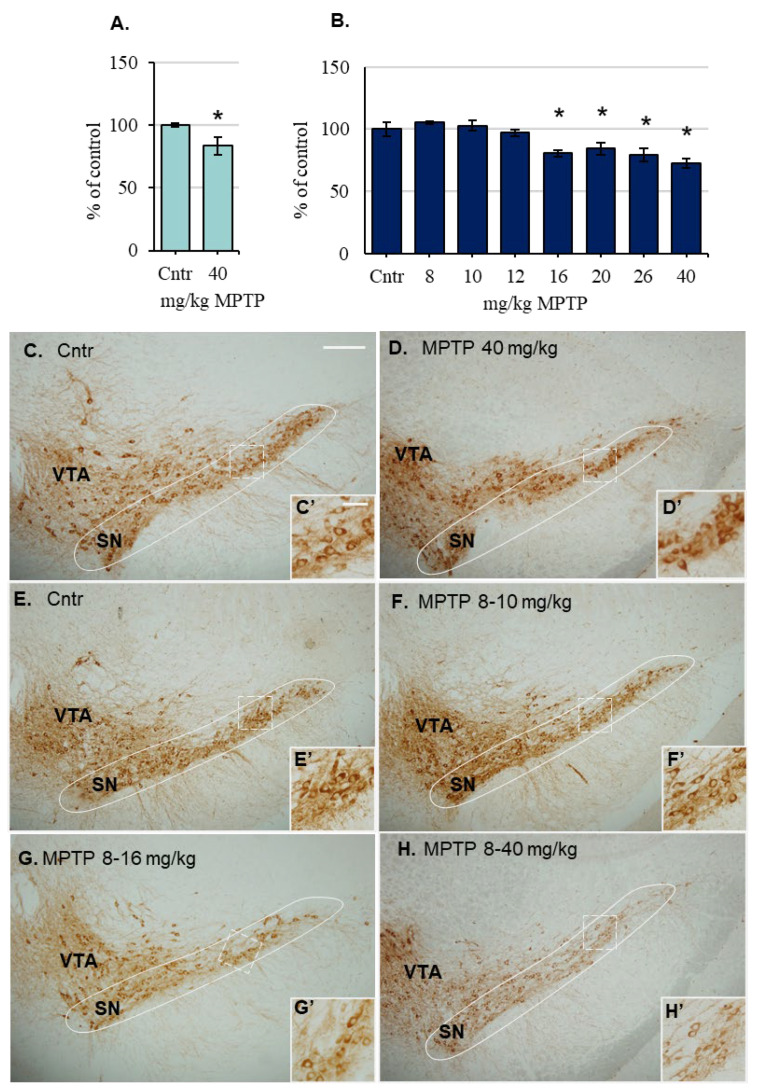
The number of tyrosine hydroxylase (TH)-immunopositive neurons (**A**) and their distribution in the substantia nigra (SN) 24 h after a single injection of 0.9% NaCl (cntr) (**C**) or 1-methyl-4-phenyl-1,2,3,6-tetrahydropyridine (MPTP) at a dose of 40 mg/kg (**D**), as well as the number of TH-immunopositive neurons in the SN 24 h after successive MPTP injections at increasing doses from 8 to 40 mg/kg (**B**) and their distribution after successive MPTP injections at doses from 8 to 10 mg/kg (**F**), from 8 to 16 mg/kg (**G**), and from 8 to 40 mg/kg (**H**), as well as after 0.9% NaCl injections (cntr) (**E**) (“n” per group = 4–6). (**C’**–**H’**) is an enlarged fragment (dashed frame) of photographs (**C**–**H**), respectively * *p* < 0.05, significant differences compared with the control, taken as 100% (Mann–Whitney U-test). Data are presented as mean ± SEM. Bars: (**C**–**H**)—200 µm; (**C’**–**H’**)—50 µm. Cntr—the control group received 0.9% NaCl; VTA—ventral tegmental area.

24 h after a single MPTP injection at a dose of 40 mg/kg, the number of neurons in the SN in the 1st group of animals decreased by 16% compared with the control (Figure 6A,C,D). In mice of the 2nd group, with repeated administration of MPTP at increasing doses, the number of neurons did not change until MPTP was injected at a dose of 12 mg/kg (Figure 6B,E,F). After the next MPTP administration at a dose of 16 mg/kg, the number of TH-immunopositive neurons decreased by 20% (Figure 6B,G) and remained at the same level with subsequent MPTP injections at doses of 20 mg/kg, 26 mg/kg, and 40 mg/kg, reaching a level of 72% after the last MPTP injection (Figure 6B,H).

## 3. Discussion

For decades, PD has been modeled on various animals, from drosophila and worms to non-human primates. However, most often PD is modeled in rats with 6-OHDA and in mice with MPTP, with specific neurotoxins of DAergic neurons. The mouse MPTP model of PD has a number of important advantages over other PD models. MPTP easily penetrates the blood–brain barrier and is converted in the brain into 1-methyl-4-phenylpyridinium (MPP^+)^, a toxin that causes dose-dependent death of DAergic neurons in the SN [28]. Previous studies have shown that the effect of MPTP on DAergic neurons is species-specific. It is effective in the brains of mice and non-human primates, but not effective in the brains of rats [38]. Although modeling of PD is preferred in monkeys, such studies are extremely rare because monkeys are not readily available for ethical and financial reasons. Modeling of PD in mice using MPTP makes it possible to reproduce the key elements of the pathogenesis and manifestations of this disease: (i) bilateral degeneration of DAergic neurons in the SN; (ii) bilateral DAergic deafferentation of the striatum; (iii) the appearance of motor disorders with a decrease in the level of DA in the striatum by 70% and the death of some DAergic neurons in the SN. Moreover, the MPTP model reproduces the systemic pathology in PD, represented by degradation, in addition to DAergic neurons of the SN, and other central and peripheral neurons [28,39,40].

Developing a new subchronic model of the progressive development of PD from the preclinical stage to the clinical stage, we were guided by the requirements for this model based on the reference points of pathogenesis and clinical manifestations in PD patients. These requirements included: (i) a decrease in the level of DA in the striatum due to the degradation of nigral neuron DAergic axons [41]; (ii) the appearance of motor disorders with a 70–80% decrease in the level of DA in the striatum [4]; (iii) loss of DAergic neurons in the SN, at least in a clinical stage model [6]; and (iv) permanent progression of the neurodegenerative process in the nigrostriatal system. To meet these requirements, we used MPTP, which is converted in the brain into the selective toxin of DAergic MPP^+^ [42,43]. To date, many different approaches have been developed to mimic PD in mice using MPTP for acute [14,15,28], subchronic [13,24,27,34,35,36], and chronic [17,18,19,20,21,22,23,26,30,31,32,33] toxin administration. These models could make it possible to study compensatory processes, and to test neuroprotectors, but they do not fully reproduce the above requirements: specifically, the continuous progressive neurodegeneration of nigral DAergic neurons. This is due to the fact that researchers developing subchronic models and chronic models of PD do not take into account the different resistance levels of individual nigral DAergic neurons to MPTP. However, an increase in the resistance of DAergic neurons to the same doses of MPTP was shown when using acute and chronic administration of a neurotoxin [29,31,43,44]. Although the mechanisms of such resistance are still poorly understood, it can be assumed that the resistance of DAergic neurons to the permanent action of MPTP increases as DAtransporter (DAT) expression decreases [45,46]. The resistance of DAergic neurons to neurotoxin could be also determined by their own compensatory processes. For example, nigral DAergic neurons expressing Ca^2+^-binding proteins calmodulin and calbindin have increased resistance in PD patients and MPTP-treated animals [47,48,49,50]. This means that in order to develop a model of PD with gradually progressive neurodegeneration of DAergic neurons in the nigrostriatal system, it is necessary to repeatedly administer MPTP at gradually increasing doses.

Based on the above idea and our previous experience [28], we used MPTP at a dose of 8 mg/kg for the first injection. According to our previous study, this was the minimum dose that led to the first manifestations of degradation of the nigrostriatal DAergic system: a decrease in the level of DA in the striatum [28]. In this study, we used 40 mg/kg as the maximum dose of MPTP, since at this dose all three signs of the clinical stage of PD were reproduced in our previous study: a decrease in the level of DA in the striatum by at least 70%, loss of DAergic neurons in the SN, and motor disorders. As intermediate doses of MPTP, doses of 10, 12, 16, 20, and 26 mg/kg were selected. This made it possible to model the gradual progressive degradation of the nigrostriatal DAergic system.

In addition to the choice of MPTP doses, the optimal interval between MPTP injections and between the last MPTP injection and obtaining materials for analysis was important for modeling the progressive degradation of the nigrostriatal DAergic system. It seems reasonable to determine this interval by the time of elimination of MPTP/MPP^+^ from the brain and the body as a whole, which occurs within approximately 24 h. Indeed, only trace amounts of MPTP/MPP^+^ have been shown to remain in the striatum 24 h after MPTP administration [23,31]. Increasing the interval between MPTP injections to more than 24 h brings about a cyclical development of reparative (compensatory) processes after the completion of neurodegenerative processes. As a result, this leads to repeated transitions of the nigrostriatal system from neurodegeneration to compensation and vice versa, which contradicts the concept of the pathogenesis of PD. Indeed, in PD, degradation of the nigrostriatal system and compensatory processes develop in parallel [51,52,53]. In reality, in previous studies, subchronic models of PD were developed with daily administration of MPTP, but at the same dose, which cannot lead to progressive degeneration of nigral DAergic neurons [20,21,32,34,35,36,50]. Moreover, the vast majority of biological samples for analysis were obtained from animals long after the last MPTP injection (from a week to a month), when compensatory processes predominate [21,32,34,35,36,50]. In chronic modeling of PD, in the most of studies, MPTP was also administered at significant intervals (from 3.5 to 7 days), which also did not lead to progressive degeneration of DAergic neurons [31,37], with the exception of a few studies using osmotic pumps with MPP^+^ [23,26,30]. However, the use of osmotic pumps for modeling PD has serious drawbacks. Therefore, on the one hand, the reproducibility of the results obtained by this method is unsatisfactory [23,26]. On the other hand, the changes observed in the nigrostriatal system in these mice do not correspond to the changes in the nigrostriatal system in PD patients. For example, in this PD model, the death of nigral DAergic neurons is not accompanied by DAergic denervation of the striatum and does not lead to motor disorders [26]. It follows from the above that this approach cannot reproduce all the critical hallmarks of pathogenesis and functional changes characteristic of PD patients.

According to our data, the most sensitive indicator of the degradation of the nigrostriatal DAergic system is the change in the DA level in the striatum, which decreases in our subchronic PD model already after the first injection of MPTP, and after the third injection it reaches a plateau of 30%. After the fourth administration of MPTP, changes also appear in the SN: the content of DA and the number of nigral DAergic neurons decrease. The loss of DAergic neurons was the same at 24 h and 5 days after the last MPTP injection and amounted to 72 and 68%, respectively (Figure S1). After the last injection of MPTP (40 mg/kg), the mice showed impaired motor behavior (Figure 1B–D) (Table 1). These data show a retrograde spread of the neurodegenerative process in the nigrostriatal system, accompanied over time by the transition from the preclinical to the clinical stage.

Unfortunately, it is basically impossible to validate models of the preclinical stage of PD in PD patients due to the lack of preclinical diagnosis of PD and, hence, pathologic studies in PD patients. Therefore, the scheme of MPTP administration, reproducing the preclinical stage of PD, was chosen on the basis of two reference points: (i) a less than 70% decrease in the level of DA in the striatum, (ii) the absence of motor disorders. These reference points were reproduced in mice with two consecutive injections of MPTP at doses of 8 and 10 mg/kg, resulting in a loss of 50% DA in the striatum, while only a decrease in DOPAC was found in the SN in these mice (Table 1).

According to the generally accepted concept of PD development, the death of nigrostriatal DAergic neurons is accompanied by compensatory processes. This explains the fact that, PD develops for a long time at the preclinical stage without the manifestation of motor symptoms [53,54]. One of these compensatory processes may be represented by increased DA neurotransmission, which is determined by the so-called DA turnover. In our study, this indicator was calculated for the striatum in two ways. In the first case, we calculated the DA turnover as the ratio of DOPAC to DA, and in the second case, as the ratio of all DA metabolites (DOPAC, HVA, 3-MT) to DA. Both indicators increase as neurodegeneration progresses in the nigrostriatal system, reaching 162% and 216%, respectively, after the 6th injection of MPTP at a dose of 26 mg/kg. After the 7th injection of MPTP, a decrease in DA turnover in relation to the control level is detected, but only according to the first indicator (DOPAC/DA), at the same time, between the 6th and 7th injections, a tendency to change was shown (*p* = 0.0833 by one-way ANOVA test). This is considered as the first manifestation of the depletion of compensatory processes during the transition of PD from the preclinical stage to the clinical stage. At the same time, this phenomenon can be considered as one of the triggers for the appearance of movement disorders. Thus, on the developed subchronic model of the preclinical and clinical stages of PD, we have been able to reproduce not only neurodegeneration, but also neuroplasticity in the form of an increase in the efficiency of DA neurotransmission.

We believe that compensatory processes in PD extend far beyond the increase in DA neurotransmission [52,53]. Indeed, with successive injections of MPTP at increasing doses, starting with a dose of 12 mg/kg, the degradation of the nigrostriatal DAergic system slows down. Therefore, in order to reproduce the progressive degradation of the nigrostriatal system, after the third MPTP injection, we had to enhance the increase in the MPTP dose for each subsequent injection. Thus, the dose of MPTP during successive injections increased by 2 mg/kg, 4 mg/kg, 6 mg/kg, and 12 mg/kg. Moreover, it seems that the resistance of neurons to MPTP increases with its repeated administration, which was also noted by us and other researchers [29,31]. To test this assumption, we compared the motor behavior and the state of the nigrostriatal system in mice in a subchronic model where MPTP was administered at increasing doses from 8 to 40 mg/kg (total dose = 132 mg/kg), on the one hand, and in mice after a single injection of MPTP at a dose of 40 mg/kg, on the other hand. Surprisingly, both treatments of mice led almost to the same changes in the main assessed indicators: motor behavior, DA concentration in the striatum, DA content in the SN, and the number of nigral DAergic neurons. Additionally, in mice with a single administration of MPTP, in contrast to mice with subchronic PD modeling, a small lethality (2 out of 12 mice died) was observed. These studies open up broad prospects for evaluating both compensatory mechanisms aimed at increasing the functional activity of surviving DAergic neurons and mechanisms for increasing the resistance of these neurons to neurotoxins.

## 4. Materials and Methods

### 4.1. Animals

Male C57BL/6 mice aged 8–12 weeks and weighing 22–25 g (n = 148) were used in this study. The animals were housed at 21–23 °C in a 12 h light–dark cycle with free access to food and water. Experimental procedures were carried out in accordance with the National Institutes of Health Guide for the Care and Use of Laboratory Animals (8th edition, 2011) and were approved by the Animal Care and Use Committee of the Koltzov Institute of Developmental Biology of the Russian Academy of Sciences (protocol №50 from 5 August 2021).

### 4.2. Experiments

**Figure 7:**
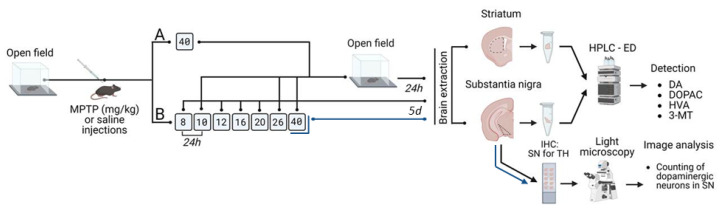
Design of experiments with subcutaneous administration of 1-methyl-4-phenyl-1,2,3,6-tetrahydropyridine (MPTP) to mice: (**A**) once at a dose of 40 mg/kg, (**B**) 7 times at gradually increasing doses from 8 to 40 mg/kg, with a preliminary analysis of motor behavior and subsequent analysis of motor behavior and the state of the nigrostriatal dopaminergic system 24 h after the last injection. 3-MT—3-methoxytyramine; DA—dopamine; DOPAC—3,4-dihydroxyphenylacetic acid; HVA—homovanillic acid; IHC—immunohistochemistry; SN—substantia nigra; TH—tyrosine hydroxylase.

We used two experimental groups of mice, which were subcutaneously injected with MPTP (Sigma-Aldrich, St. Louis, MO, USA), as well as two control groups of mice, which were subcutaneously injected with 0.9% NaCl according to the same regime. Materials for analysis were obtained 24 h after the last injection (Figure 7).

In the first group, mice (n = 12) received MPTP once at a dose of 40 mg/kg (Figure 7A). In the second group, mice were successively injected with MPTP at increasing doses of 8 (n = 6), 10 (n = 10), 12 (n = 6), 16 (n = 6), 20 (n = 6), 26 (n = 10), and 40 mg/kg (n = 12) at 24 h intervals (Figure 7B). Material for analysis was obtained 24 h after each injection. Additionally, mice that were successively injected with MPTP at increasing doses from 8 to 40 mg/kg (n = 6) were decapitated 5 days after the last injection of MPTP to count neurons containing TH (Figure S1). The same number of animals was used in each control group (administration of 0.9% NaCl).

In mice that were injected with MPTP once at a dose of 40 mg/kg, or twice at doses of 8 and 10 mg/kg, 6 times at MPTP doses from 8 to 26 mg/kg, or 7 times at MPTP doses from 8 to 40 mg/kg, as well as in the control mice injected with 0.9% NaCl, motor behavior was preliminarily assessed using an automated PhenoMaster device (TSE Systems, Germany) with software. These mice were assessed for 6 min in the open field test for traveled distance, as well as for the number of fine movements and rearings. According to the characteristics obtained, the mice were divided into experiment and control groups so that the average distance traveled in the open field test was the same. Twenty three and a half hours after the last injection of MPTP or 0.9% NaCl, motor behavior was reassessed in all animals in the open field.

### 4.3. Sample Preparation for Analysis

Mice from all experimental and control groups were decapitated under isoflurane anesthesia (Baxter, Deerfield, IL, USA), the brains were removed and cut along the middle sagittal plane. The striatum was isolated from one cerebral hemisphere from bregma 1.70 to bregma 0.14 in the rostrocaudal direction according to the atlas [55] and the SN was isolated from bregma −2.54 to bregma −4.04 using a dissecting microscope (Leica M60, Wetzlar, Germany). This procedure was described in detail earlier [28,39]. The obtained samples from mice of the 1st and 2nd groups of animals (n = 6–8) were weighed, frozen in liquid nitrogen, and stored at −70 °C until the concentration of DA and its metabolites was determined by HPLC-ED.

The second hemisphere of the brain, obtained from mice of the 1st and 2nd groups of animals (n = 4–6 per group) in the experiment and in the control, was fixed by immersion in 4% paraformaldehyde in 0.2 M phosphate buffer (pH 7.2–7.4) for 12 h at 4 °C. The brain was then washed with 0.02 M phosphate-buffered saline (PBS) (pH 7.2–7.4) at room temperature and incubated in PBS with 20% sucrose at 4 °C for 12 h (all reagents from Sigma-Aldrich, St. Louis, MO, USA). The brain was then frozen in hexane at −40 °C and stored at −70 °C until immunostaining for TH.

### 4.4. Methods

#### 4.4.1. High Performance Liquid Chromatography with Electrochemical Detection

HPLC-ED was used to determine the concentration of DA, DOPAC, HVA, and 3-MT in the striatum samples of mice from groups 1 and 2, as well as to determine the content of DA, DOPAC, and HVA in the SN samples (Figure 7). The samples were homogenized using an ultrasonic homogenizer (UP100H, Hielscher Ultrasonics GmbH, Teltow, Germany) in 0.1 N HClO_4_ (Sigma-Aldrich, St. Louis, MO, USA) in a solution containing the internal standard 3,4-dihydroxybenzylamine hydrobromide (Sigma-Aldrich, St. Louis, MO, USA) at a concentration of 250 pmol/mL. After that, the solution was centrifuged at 2000× *g* for 20 min.

The separation of DA and its metabolites was carried out on a ReproSil-Pur reversed-phase column, ODS-3, 4 × 100 mm with a pore diameter of 3 µm (Dr. Majsch, Ammerbuch, Germany) at a temperature of +30 °C and a mobile phase speed of 1 mL/min, supported by an LC-20ADsp liquid chromatograph (Shimadzu, Kyoto, Japan). The mobile phase included: 0.1 M citrate-phosphate buffer, 0.3 mM sodium octanesulfonate, 0.1 mM EDTA, and 9% acetonitrile (all reagents from Sigma-Aldrich, St. Louis, MO, USA), pH 2.5. A Decade II electrochemical detector (Antec Leyden, Leuden, The Netherlands) was equipped with a working glassy carbon electrode (+0.85 V) and an Ag/AgCl reference electrode. Peaks of DA, DOPAC, HVA, 3-MT and the internal standard were identified by their release time in the standard solution. The content of analytes was calculated by the internal standard method as the ratio of the peak areas of DA and its metabolite standards to the peak areas of these substances in a biological sample using the LabSolutions software (Shimadzu, Japan). Striatum samples were normalized to tissue weight.

#### 4.4.2. Immunohistochemistry

From the frozen brain of mice from groups 1, 2, and 3 in the experiment and in the control, serial frontal 20 μm thick sections of the SN were made along its entire length in the rostrocaudal direction, from bregma −2.54 to bregma −4.04 according to the atlas [52] (Figure 7), using a cryostat (Leica CM1950, Germany). Every 6th section was mounted on a glass slide. The sections were then consecutively incubated with: (i) 0.03% H_2_O_2_ in PBS saline for 30 min; (ii) 3% bovine serum albumin (Sigma-Aldrich, St. Louis, MO, USA) and 0.3% Triton-X100 (Sigma-Aldrich, St. Louis, MO, USA) in PBS for 30 min; (iii) sheep antibodies against TH (1:700) (ab1542, Millipore, Burlington, MA, USA), 1% bovine serum albumin, and 0.1% Triton X-100 in PBS for 20 h; (iv) biotinylated anti-sheep IgG Reagent (1:200) (Vector Laboratories, Burlingame, CA, USA) in PBS for 2 h; and (v) avidin-biotin peroxidase complex (Vector Laboratories, USA) in PBS for 1 h. After each incubation, except for that prior to the incubation with primary antibodies (point iii), the sections were washed in PBS for 30 min. Peroxidase of the avidin-biotin complex was detected in PBS with 0.05% 3,3′-diaminobenzidine tetrahydrochloride (Sigma-Aldrich, St. Louis, MO, USA) and 0.02% H_2_O_2_ under dissecting microscope control (Leica M60, Germany). All incubations were carried out at +20 °C. After the described procedures, the sections were embedded into a Mowiol hydrophilic medium (Sigma-Aldrich, St. Louis, MO, USA).

#### 4.4.3. Microscopy and Image Analysis

The analysis of SN sections and photography of TH-immunopositive neurons were performed using an Olympus BX51 light microscope (Olympus, Japan) equipped with an Olympus DP70 digital camera (Olympus, Japan), with a 10× and 40× objective magnification. The cell bodies of only those immunostained neurons in which the nucleus was visible were counted using the FiJi software (available online: (https://imagej.net/software/fiji/downloads) accessed on 10 August 2022). The approximation method was used to determine the total number of DAergic neurons in the SN [56] (Figure 7).

#### 4.4.4. Statistical Analysis

Statistical processing of the obtained results was carried out by one-way ANOVA, the parametric Student’s *t*-test, or the non-parametric Mann–Whitney U-test using the GraphPad Prism 6.0 software package (GraphPad Software, La Jolla, CA, USA). *p* ˂ 0.05 was considered to be a significant difference; *p* < 0.1 was considered as a tendency to difference.

## 5. Conclusions

The fight against neurodegenerative diseases, including PD, is among the challenges of the 21st century. The low effectiveness of PD treatment is due to the fact that it is diagnosed by the appearance of motor symptoms and begins to be treated only many years after the onset. A key link in the pathogenesis of PD is the death of DAergic neurons of the nigrostriatal system of the brain responsible for the regulation of motor function. By the time the first motor symptoms appear, the level of DA in the striatum, the site of DAergic axon projection, decreases by 70–80%. One of the most advanced strategies for combating PD is to develop early (preclinical) diagnosis and preventive neuroprotective treatment that slows down neuronal death. In accordance with the paradigm of translational medicine, the development of such technologies should be based on fundamental knowledge about the molecular mechanisms of PD pathogenesis. Since obtaining biological samples from patients is problematic at the clinical stage, and even impossible at the preclinical stage, most studies of the molecular mechanisms of PD pathogenesis are carried out on experimental models. Specifically, in this study, we have developed a subchronic mouse model of PD by repeated injections of MPTP, a toxin of DAergic neurons, at gradually increasing doses and with a 24 h interval between injections comparable with the time of MPTP clearance. This model reproduces the main features characterizing the development of PD: (i) progressive death of DAergic neurons and denervation of the striatum; (ii) a compensatory increase in DA turnover and hence the efficiency of DA neurotransmission; and (iii) the appearance of motor disorders due to the loss of 70–80% of DA in the striatum, which in patients leads to the transition of PD from the preclinical stage to the clinical one.

## Figures and Tables

**Table 1:** The main characteristics of the developed models of Parkinson’s disease: at the preclinical stage and at the clinical stage by daily administration of MPTP in mice at a gradually increasing dose from 8 mg/kg to 10 mg/kg for 2 days and from 8 mg/kg to 40 mg/kg for 7 days, respectively, and at the clinical stage by a single MPTP administration at a dose of 40 mg/kg.

	Subchronic Models		Acute Model
	Preclinical Stage	Clinical Stage	Clinical Stage
Daily MPTP injections (mg/kg)	8, 10	8, 10, 12, 16, 20, 26, 40	40
Motor behavior	No motor disorders	Motor disorders	Motor disorders
DA level in the striatum compared to the control (100%)	47%	26%	25%
Number of DAergic neurons in the SN compared to the control (100%)	97%	72%	84%
Dopamine turnover (DOPAC/DA) as an indicator of neurotransmission efficiency	Increased	Decreased	Increased

## Data Availability

The data presented in this study are available on request from the corresponding author. The data are not publicly available due to legal issues.

## Supplementary Materials

The following supporting information can be downloaded at: https://www.mdpi.com/article/10.3390/ijms24010683/s1.

## Abbreviations

3-MT	3-methoxytyramine
AADC	aromatic L-amino acid decarboxylase
DA	dopamine
DAT	dopamine transporter
DOPAC	3,4-dihydroxyphenylacetic acid
HVA	homovanillic acid
MPP+	1-methyl-4-phenylpyridinium
MPTP	1-methyl-4-phenyl-1,2,3,6-tetrahydropyridine
PBS	phosphate-buffered saline
PD	Parkinson’s disease
SN	substantia nigra
TH	tyrosine hydroxylase
