# Supplementary material for: Modeling of the Progressive Degradation of the Nigrostriatal Dopaminergic System in Mice to Study the Mechanisms of Neurodegeneration and Neuroplasticity in Parkinson’s Disease
Source: Int J Mol Sci. 2022 Dec 30;24(1):683. doi: 10.3390/ijms24010683 (PMC9820573; doi:10.3390/ijms24010683)
Supplement: Supplementary file 1 [file ijms-24-00683-s001.zip › ijms-2069532-supplementary.pdf]

Supplementary Table S1. The concentrations of dopamine, 3,4-dihydroxyphenylacetic acid (DOPAC), homovanillic acid (HVA), and 3-methoxytyramine (3-MT) in the striatum of mice 24 hours after a single injection of 1-methyl-4-phenyl-1,2,3,6-tetrahydropyridine (MPTP) at a dose of 40 mg/kg or after each of the successive injections of 1-methyl-4-phenyl-1,2,3,6-tetrahydropyridine (MPTP) at increasing doses from 8 to 40 mg/kg. Control group received 0.9% NaCl according to the same schemes.

**Group, 0,9% NaCl or MPTP (mg/kg)**	**Dopamine, pmol/mg**	***p****	**DOPAC, pmol/mg**	***p****	**HVA, pmol/mg**	***p****	**3-MT, pmol/mg**	***p****
**Single injection of MPTP**								
Control	103.1 ± 2.0		6.3 ± 0.4		12.0 ± 0.6		2.0 ± 0.2	
40mg/kg	25.4 ± 3.3	*1.5E-09*	2.7 ± 0.2	*7.13E-05*	8.1 ± 0.7	*1.93E-03*	1.1 ± 0.1	*2.77E-03*
**Successive injections of MPTP**								
Control	110.4 ± 3.6		9.4 ± 0.2		9.2 ± 0.2		3.1 ± 0.3	
8	91.9 ± 1.9	*1.1E-03*	8.1 ± 0.4	*0.02*	8.7 ± 0.5	*0.39*	2.9 ± 0.1	*0.58*
8-10	51.8 ± 3.0	*1.8E-07*	5.3 ± 0.2	*3.5E-07*	7.4 ± 0.6	*0.03*	2.3 ± 0.2	*0.08*
Control	102.1 ± 2.3		6.4 ± 0.4		10.1 ± 1.0		2.7 ± 0.3	
8-10-12	34.1 ± 1.5	*3.0E-07*	3.0 ± 0.1	*1.9E-04*	5.5 ± 0.3	*1.6E-03*	1.8 ± 0.2	*0.04*
Control	103.2 ± 2.8		8.8 ± 0.2		10.4 ± 0.2		2.7 ± 0.4	
8-10-12-16	31.0 ± 1.6	*7.2E-10*	3.6 ± 0.2	*2.1E-08*	6.2 ± 0.5	*2.6E-05*	1.8 ± 0.3	*0.13*
Control	98.8 ± 1.9		7.3 ± 0.3		9.8 ± 0.2		3.1 ± 0.1	
8-10-12-16-20	35.1 ± 3.0	*5.7E-09*	3.9 ± 0.3	*1.5E-05*	8.2 ± 0.5	*0.01*	3.4 ± 0.2	*0.17*
Control	107.5 ± 3.6		6.0 ± 0.4		7.6 ± 0.5		3.1 ± 0.4	
8-10-12-16-20-26	29.1 ± 1.0	*4.1E-12*	2.5 ± 0.3	*0.01*	5.3 ± 0.5	*0.01*	2.0 ± 0.2	*6.9E-01*
Control	104.6 ± 3.3		7.9 ± 0.2		9.2 ± 0.1		1.6 ± 0.2	
8-10-12-16-20-26-40	26.7 ± 1.2	*6.0E-12*	2.7 ± 0.3	*5.1E-09*	6.4 ± 0.7	*0.01*	0.8 ± 0.1	*2.04E-04*

p* calculated relative to the control group.

Supplementary Table S2. The content of dopamine, 3.4-dihydroxyphenylacetic acid (DOPAC) and homovanillic acid (HVA) in the substantia nigra of mice 24 hours after a single injection of 1-methyl-4-phenyl-1,2,3,6-tetrahydropyridine (MPTP) at a dose of 40 mg/kg or after each of the successive injections of 1-methyl-4-phenyl-1,2,3,6-tetrahydropyridine (MPTP) at increasing doses from 8 to 40 mg/kg. Control group received 0.9% NaCl according to the same schemes.

**Group. 0.9% NaCl or MPTP (mg/kg)**	**Dopamine. pmol**	*p**	**DOPAC. pmol**	*p**	**HVA. pmol**	*p**
**Single injection of MPTP**						
Control	8.9 ± 0.5		2.8 ± 0.2		4.1 ± 0.2	
40	7.0 ± 0.6	*0.03*	1.9 ± 0.2	*0.02*	3.9 ± 0.2	*0.43*
**Successive injections of MPTP**						
Control	6.9 ± 0.7		2.6 ± 0.2		3.7 ± 0.2	
8	7.5 ± 0.5	*0.5*	2.1 ± 0.1	*0.03*	4.5 ± 0.3	*0.051*
8-10	6.8 ± 0.3	*0.94*	2.3 ± 0.1	*0.23*	4.7 ± 0.5	*0.11*
Control	6.2 ± 0.3		2.3 ± 0.1		3.3 ± 0.3	
8-10-12	5.5 ± 0.4	*0.18*	1.7 ± 0.0	*0.005*	2.8 ± 0.2	*0.13*
Control	7.3 ± 0.4		2.1 ± 0.2		3.2 ± 0.2	
8-10-12-16	5.8 ± 0.4	*0.03*	1.7 ± 0.2	*0.08*	2.8 ± 0.2	*0.22*
Control	8.2 ± 0.4		2.1 ± 0.1		3.3 ± 0.2	
8-10-12-16-20	4.4 ± 0.2	*4.9E-05*	1.5 ± 0.1	*3.7E-04*	3.0 ± 0.1	*0.30*
Control	7.1 ± 0.3		2.4 ± 0.2		2.4 ± 0.1	
8-10-12-16-20-26	7.1 ± 0.3	*8.4E-04*	1.2 ± 0.2	*1.1E-04*	2.0 ± 0.2	*0.07*
Control	7.8 ± 0.5		2.4 ± 0.2		3.4 ± 0.2	
8-10-12-16-20-26-40	4.3 ± 0.3	*2.1E-05*	1.0 ± 0.1	*1.0E-08*	3.5 ± 0.2	*0.76*

p* calculated relative to the control group.

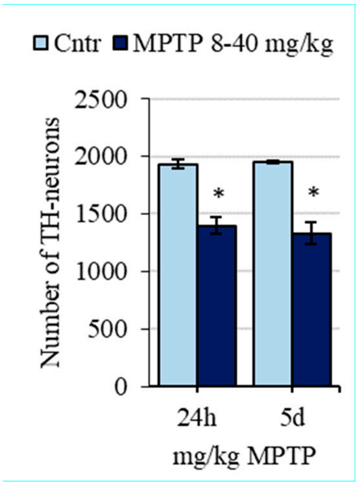

Figure S1. The number of tyrosine hydroxylase (TH)-immunopositive neurons in the substantia nigra 24 hours and 5 days after successive MPTP injections at increasing doses from 8 to 40 mg/kg ("n" per group = 5). *$p<0.05$, significant differences compared with the control (Mann-Whitney U-test).
